# *Tieguanyin* Oolong Tea Extracts Alleviate Behavioral Abnormalities by Modulating Neuroinflammation in APP/PS1 Mouse Model of Alzheimer’s Disease

**DOI:** 10.3390/foods11010081

**Published:** 2021-12-29

**Authors:** Hyunuk Kang, Hui Zhou, Yushan Ye, Jiangfan Yang, Zhonghua Liu, Puming He, Bo Li, Yuanyuan Wu, Yaomin Wang, Youying Tu

**Affiliations:** 1 Department of Tea Science, Zhejiang University, Hangzhou 310058, China; 11616111@zju.edu.cn (H.K.); 3150102861@zju.edu.cn (H.Z.); ysye@zju.edu.cn (Y.Y.); larkin-liu@163.com (Z.L.); pmhe@zju.edu.cn (P.H.); drlib@zju.edu.cn (B.L.); yywu@zju.edu.cn (Y.W.); 2 Key Laboratory of Tea Science, College of Horticulture, Fujian Agriculture and Forestry University, Fuzhou 350002, China; yjf3001@163.com; 3 Key Laboratory of Tea Science of Ministry of Education, Hunan Agricultural University, Changsha 410128, China; 4 Key Laboratory of Horticulture Plant Biology, Ministry of Education, College of Horticulture and Forestry Sciences, Huazhong Agricultural University, Wuhan 430070, China

**Keywords:** oolong tea, chemical components, oxidative stress, inflammation, cognitive impairment

## Abstract

Alzheimer’s disease (AD) is a common neurodegenerative disease; tea components have important neuroprotective effects. This article explores the effects and mechanisms of Qingxiang *Tiguanyin* (Tgy-Q), Nongxiang *Tieguanyin* (Tgy-N), and Chenxiang *Tieguanyin* (Tgy-C) extracts on APP/PS1 AD model mice. Morris water maze and new object recognition experiments show that *Tieguanyin* extracts can effectively enhance the cognitive ability of APP/PS1 mice. H&E staining, Nissl staining, and immunohistochemical staining show that *Tieguanyin* extracts make nerve cell boundaries and nucleoli become clearer, relieve nucleus pyknosis, and effectively reduce Aβ1-40 and Aβ1-42 in the hippocampus and cortex. They also restore the morphology of microglia and astrocytes. In addition, *Tieguanyin* extracts can balance the oxidative stress level in the brain of APP/PS1 mice by improving the antioxidant capacity. Western blot results show that *Tieguanyin* extracts can reduce the expression of NF-κB p65, TNF-α, IL-1β, IL-6, COX-2, and iNOS in mouse brain, which demonstrates that *Tieguanyin* extracts improves cognitive ability by alleviating inflammation. This article demonstrates for the first time that *Tieguanyin* extracts can inhibit the excessive activation of the NF-κB p65 signaling pathway and improve the antioxidant capacity in the cerebral cortex and hippocampus, to improve the cognitive ability of APP/PS1 mice. Our results shed light into the beneficial of *Tieguanyin* tea extracts on preventing and alleviating AD diseases.

## 1. Introduction

Alzheimer’s disease (AD) is one of the most common degenerative diseases of the central nervous system and is characterized by cognitive loss. Its main characteristics are memory dysfunction, personality mutations, impaired cognitive function, and language degradation as symptoms [1]. The 2019 annual report of Alzheimer’s Disease International (ADI) shows that more than 50 million people worldwide suffer from dementia, and the number of people affected will exceed 152 million by 2050 [2]. There are two main countermeasures against AD: one is treatment with drugs, and the other is adjuvant therapy without drugs. Gene therapy, drugs that interfere with Aβ, affect free radical metabolism, and anti-inflammatory drugs can alleviate the symptoms of AD. Donepezil, Listigmine, Galantamine and Memantine are the main drugs currently used for the treatment of AD. Donepezil is a central acetylcholinesterase inhibitor, which has improved cognitive symptoms mainly by regulating the level of acetylcholine and can be used as a positive control drug [3]. In recent years, studies have shown that healthy diet is associated with a reduction in the risk of central nervous system disease, and nutritional intervention has become a research hotspot in the prevention and treatment of nervous system diseases [4]. Functional compounds from natural products can effectively improve AD symptoms [5,6]. Tea is second the most regularly consumed drink after water in the world, and contains bioactive ingredients such as tea polyphenols, caffeine, protein, flavonoid glycosides, amino acids, vitamins, etc. Tea drinking as well as consuming tea ingredients has a positive effect on improving cognitive ability. In vitro and in vivo studies also have demonstrated the neuroprotective effects of functional tea ingredients [7,8,9]. Oolong tea is a semi-oxidized Chinese tea made by fresh tea leaves. *Tieguanyin* is a variety of Oolong tea, originally produced in Anxi County, Fujian Province. *Tieguanyin* can be divided into three categories according to different processing methods and flavors, namely, the Qingxiang *Tieguanyin* (Tgy-Q), Nongxiang *Tieguanyin* (Tgy-N) and Chenxiang *Tieguanyin* (Tgy-C). Recent studies have shown that *Tieguanyin* tea has anti-oxidant, anti-tumor, lipid-lowering, anti-inflammatory and other health benefits [10,11]. However, the neuroprotective effect of *Tieguanyin* remains unclear. This study used a APPswe/PS1dE9 (APP/PS1) double transgenic mouse model, which is extensively used for the study of biochemical and pathological mechanisms as well as exploring therapeutic treatments of AD [12]. Multiple techniques were used to explore the effect and mechanism of three different *Tieguanyin* tea extracts on AD to provide a more scientific basis for the in-depth elucidation of *Tieguanyin*’s health functions.

## 2. Results

### 2.1. Major Chemical Components in Three Different Types of Tieguanyin Extracts

**Figure 1:**
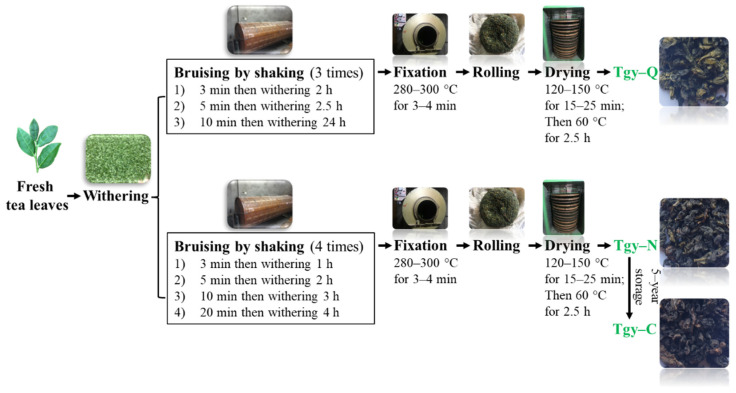
Manufacturing processes of making three types of *Tieguanyin* tea.

The detailed manufacturing processes of making Tgy-Q, Tgy-N, and Tgy-C are shown in Figure 1. After the purification process to obtain the extracts, the contents of major chemical components in these three types of *Tieguanyin* extracts were measured and shown in Table 1. All three extracts contain higher amount of bioactive ingredients, including tea polyphenol, free amino acids, soluble protein, soluble sugar, tea polysaccharides, flavonoids, caffeine, and catechins. Among which, tea polyphenol and soluble sugar are the most abundant. The content of tea polyphenol and soluble sugar in Tgy-Q is 437.25 and 362.01 mg/g, respectively; Tgy-N is 396.48 and 336.52 mg/g, respectively; Tgy-C is 368.26 and 278.18 mg/g, respectively. Among the three types of Tgy-Q, Tgy-N, and Tgy-C extracts, the levels of tea polyphenol, free amino acids, and soluble sugar were significantly decreased, while soluble proteins, tea polysaccharides, and flavonoids increased significantly. As catechins account for 60–80% of the total tea polyphenols, all three *Tieguanyin* extracts contains high amount of catechins that range from 227.78 to 294.56 mg/g. Meanwhile, ester catechins were much higher than simple catechins for all three extracts, and among the ester catechins, the content of EGCG is the highest, ranging from 77.42 to 105.21 mg/g.

### 2.2. Tieguanyin Extracts Improves Cognitive Ability in APP/PS1 Mice

**Figure 2:**
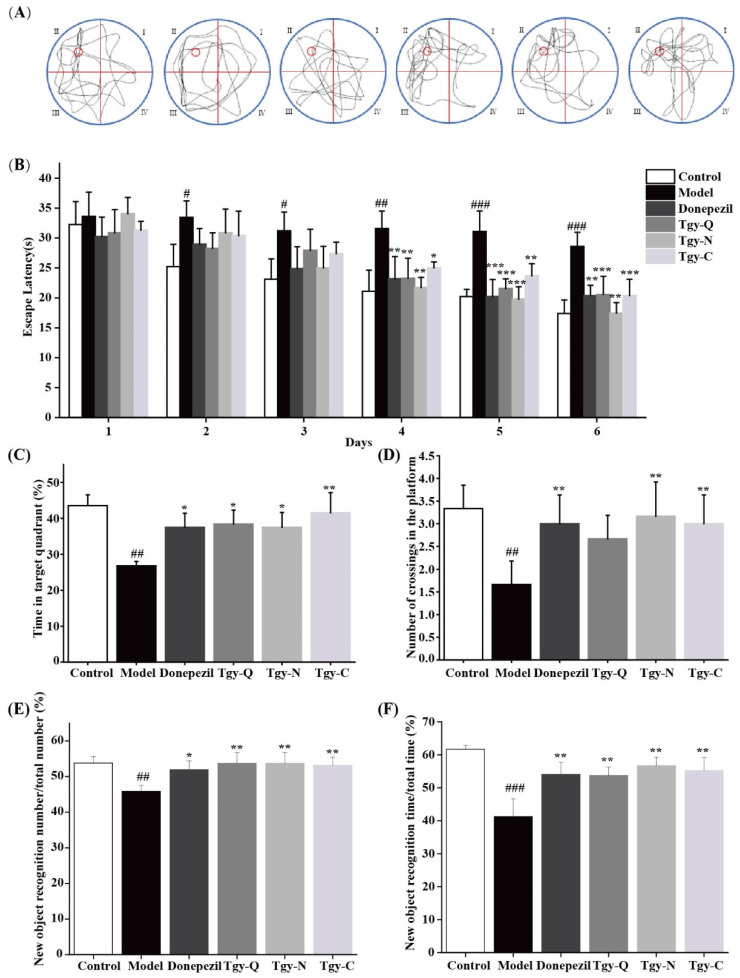
*Tieguanyin* extracts improve the cognitive ability. (**A**) Trajectory map of Morris maze in six groups of mice, the area of water maze circular pool was divided into four quadrants I, II, III, and IV, the target object was located in II area. (**B**) Escape latency of looking for invisible platform in six groups of mice. (**C**,**D**) Remaining time of target area and number of crossings in six groups of mice. (**E**,**F**) Effect of *Tieguanyin* extracts on APP/PS1 mice in new object recognition experiment. Donepezil group indicates positive control group (*n* = 8, APP/PS1 mice), daily delivered with 1 mg/kg/day Donepezil in 0.25 mL Sterile by oral gavage for 70 days; Control group (*n* = 10, C57BL/6 J mice), Model, Tgy-Q, Tgy-N, Tgy-C groups each has 8 APP/PS1 mice, which was administrated with corresponding sterile water or different extracts dissolved in 0.25 mL sterile water by daily oral gavage for 70 days. Data are mean ± SEM. # *p* < 0.05, ## *p* < 0.01, and ### *p* < 0.001 different from control group, * *p* < 0.05, ** *p* < 0.01, and *** *p* < 0.001 different from model group.

To explore the effect of *Tieguanyin* extracts on the cognitive ability of mice, the Water maze experiment was first carried out. As shown in Figure 2A, the movement trajectory of the Control group mice is close to the search platform. And the mice in the Tgy-Q, Tgy-N, and Tgy-C group mice move trajectories close to the platform position, showing obvious selectivity, suggesting that *Tieguanyin* extracts enhanced the spatial memory ability of mice.

The escape latency refers to the time it takes for the mice to find the setting platform from the time they enter the water; that is, the shorter the escape latency, the stronger the learning ability of the mice. As shown in Figure 2B, on the fourth day, compared with the model group (vehicle-treated APP/PS1 mice), the escape latency of the *Tieguanyin* extracts groups were significantly shortened, dropping to 26%, 31%, and 15%, respectively. Among them, the Tgy-N group had the best effect; on the sixth day, the escape latency of the three *Tieguanyin* treatment groups was significantly shortened (*p* < 0.01), which was reduced to 28%, 42%, and 26% of the model group, respectively. These results suggest that the treatment of *Tieguanyin* extracts by intragastric administration effectively protects the cognitive impairment of APP/PS1 mice and enhances the learning and memory ability of APP/PS1 mice. We further explored the effect of *Tieguanyin* extracts on the residence time of APP/PS1 mice in quadrant II, results showed that the time of staying in the quadrant of the platform increased to 43%, 40%, and 55% of the model group, respectively (Figure 2C). Compared with the other two groups, the mice in the Tgy-C group stayed longer in the target quadrant, which indicated that the learning and memory ability was improved. In addition, the *Tieguanyin* extracts significantly increased the number of APP/PS1 mice crossing the hidden platform. Compared with the model group, the Tgy-N and Tgy-C group mice that crossed the original platform significantly increased by 81% and 71.4%, respectively (*p* < 0.01). Compared with the model group, the number of Tgy-Q group mice that crossed the original platform increased by 52%, but there was no significant difference (*p* > 0.05). These results show that *Tieguanyin* extracts improves the space exploration ability of APP/PS1 mice.

The principle of the new object recognition experiment is that mice have a desire to detect new objects. If the cognitive function is barrier-free, the time to exploring new objects will be longer. As shown in Figure 2E, F, compared with mice in the Control group, the number of new object explorations in the model group was reduced by 14.74% (*p* < 0.01), and the time was significantly reduced by 33.1% (*p* < 0.001). Compared with the model group mice, the Donepezil group increased the number of times to explore new objects by 13.26% (*p* < 0.05), and the time was significantly increased by 30.94% (*p* < 0.01). The number and time of exploring new objects in the three *Tieguanyin* extracts treatment groups increased significantly (*p* < 0.01), and the longest time for mice in Tgy-N group to explore new objects reached 37.34%.

### 2.3. Tieguanyin Extracts Improves the Pathology of the Cortex and Hippocampus of APP/PS1 Mice

**Figure 3:**
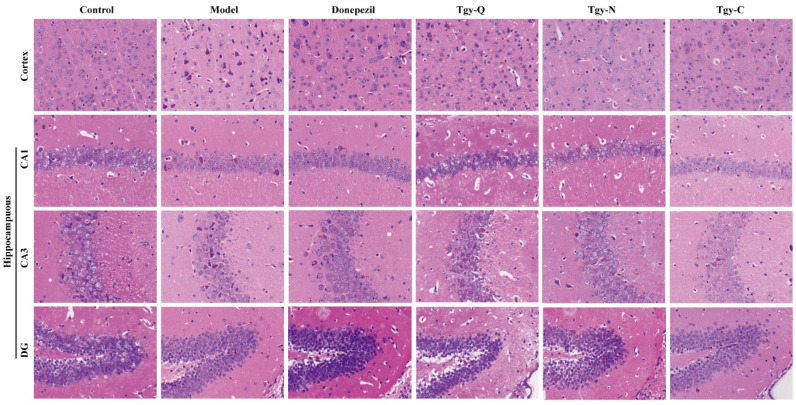
Effect of *Tieguanyin* extracts treatment on neuronal loss and death in the cortex and in the hippocampus by HE staining in the cortex and hippocampus (CA1, CA3, DG) of the wild-type mice and the APP/PS1 mice treated with normal saline, Tgy-Q, Tgy-N,Tgy-C, or Donepezil (0.1 mg/kg). *n* = 3 for each group.

From the above behavioral results, we found that the three *Tieguanyin* extracts significantly improved cognition, learning, and memory abilities of APP/PS1 mice, suggesting that it may work by improving the pathological state of the mouse brain. To confirm this hypothesis, we first observed its effect on brain tissue by H&E staining. The results show (Figure 3) that the cortex and hippocampus (CA1, CA3, DG) of the normal group of mice have uniform staining of nerve cells, normal structure, neatly arranged, and large numbers of neurons, round nuclei, obvious nucleoli, cell membranes, and nuclei. The cortex and hippocampus of the model group mice showed abnormal tissue structure, cell morphology changes, and most neurons showed pyknotic nuclei, deep staining of cytoplasm, and unclear boundaries between cell and nuclear. The number of neurons in the cortex and hippocampus of the Donepezil group mice was relatively large. The morphology was improved, the nucleus was round, and the nucleolus was obvious. The neurons in the cortex and hippocampus of the mice in the Tgy-Q group were fairly neatly arranged, and part of the nucleus was abnormally stained. The number of neurons in the cortex and hippocampus of the Tgy-N group of mice was large and neatly arranged. The neurons were large, the cell membrane was clear, the nucleus was round, and the nucleoli were obvious. The cortex and hippocampus of the mice in the Tgy-C group are arranged neatly, with round nuclei and obvious nucleoli. Occasionally, the nuclei are concentrated and deeply stained, and the cell morphology changes. Compared with the model group, the pathological conditions of the three *Tieguanyin* extracts groups are all improved.

### 2.4. Effect of Tieguanyin Extracts on Cortical Neurons of APP/PS1 Mice

**Figure 4:**
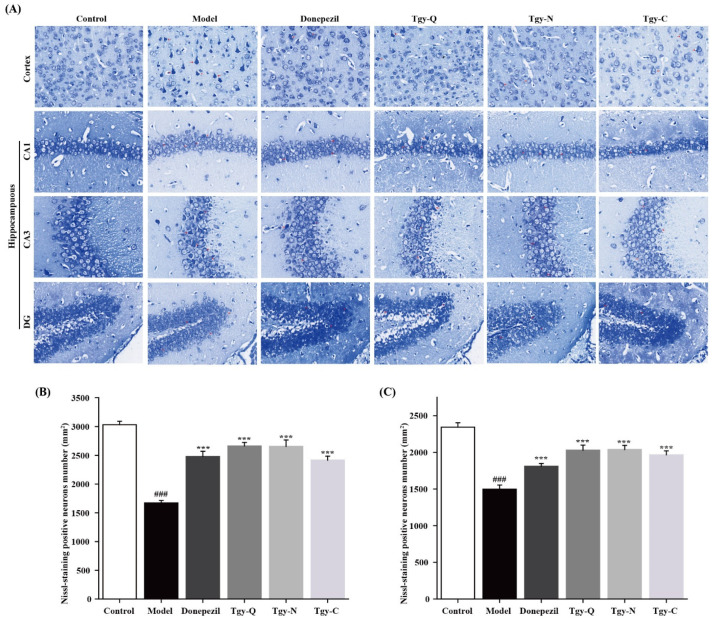
Nissl histochemical stain for Nissl bodies in the neurons of cortex and hippocampus in different mouse groups. (**A**) Nissl staining images of cortex and hippocampus (×400 magnification). (**B**) Cortical Nissl body statistics chart. (**C**) Nissl body number in hippocampus. *n* = 3 for each group. ### *p* < 0.001 different from control group. *** *p* < 0.001 different from model group.

Nissl body, as a neuron’s marker substance, its morphology and structure can reflect the neuron’s morphology and function. As shown in Figure 4A, the neurons in the cortex and hippocampus (CA1, CA3, DG) of the control group mice are arranged neatly, the cell structure is complete and uniform, the number of neurons is large, the number of Nissl bodies is large, and the morphology is normal. Compared with the normal group, the cells in the cortex and hippocampus of the model group were scattered, the number of Nissl bodies was significantly reduced (*p* < 0.001), or even disappeared, the staining became lighter, the cell morphology changed. Compared with the model group, the cells in the cortex and hippocampus of the Donepezil group are more evenly arranged, the cytoplasm is darker, the number of Nissl bodies is significantly increased (*p* < 0.001), and the cell morphology is abnormal occasionally. Compared with the model group, the cells in the cortex and hippocampus of the Tgy-Q group were neatly arranged, the number of Nissl bodies was significantly increased (*p* < 0.001), the cytoplasmic staining was deeper, and abnormal cell morphology was occasionally observed. The cells in the cortex and hippocampus of the Tgy-N group were arranged neatly, the number of Nissl bodies increased significantly (*p* < 0.001), the cytoplasm was stained darker, and some cells were abnormal. The cells in the cortex and hippocampus of the Tgy-C group were neatly arranged, the number of Nissl bodies increased significantly (*p* < 0.001), and some cells were abnormal in morphology. The pathological conditions of the three *Tieguanyin* extracts groups were improved, and the Tgy-Q group was the weakest (Figure 4B,C).

### 2.5. Tieguanyin Extracts Improves the Deposition of Aβ1-42 Amyloid Plaques in the Cortex and Hippocampus of APP/PS1 Mice

**Figure 5:**
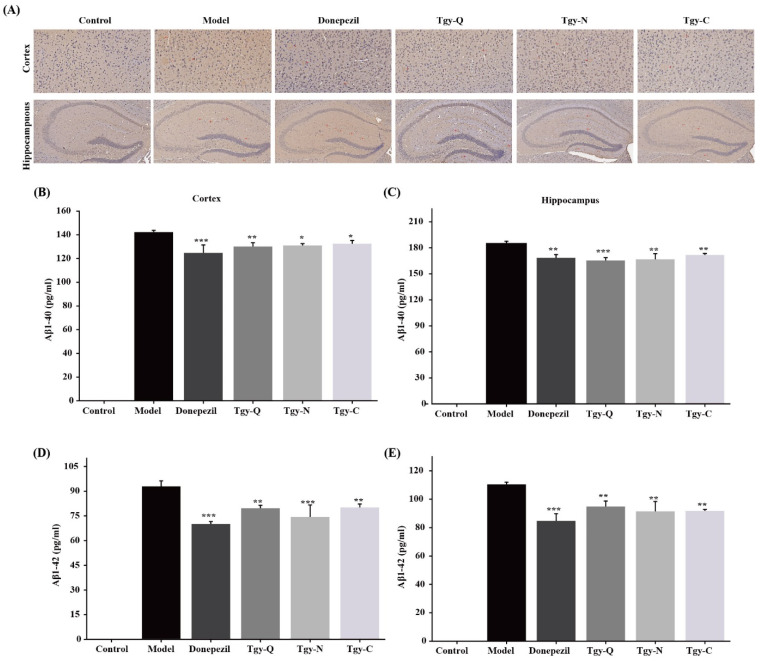
The effects of *Tieguanyin* tea extracts on expression of Aβ (1-42) in the brain of APP/PS1 transgenic mice by immunohistochemical staining. (**A**) Cortex (×200), Hippocampus (×100). *n* = 3 for each group. (**B**,**C**) Aβ1-40 and (**D**,**E**) Aβ-42 levels in the cortex and hippocampus in APP/PS1 mice (*n* = 5 for each group; control group was not detected, *n* = 7). * *p* < 0.05, ** *p* < 0.01 and *** *p* < 0.001 different from model group.

An important pathological sign of Alzheimer’s disease is the deposition of amyloid plaque Aβ in the cortex and hippocampus. Further observation of the effect of *Tieguanyin* extracts on Aβ (1-42) in the brain endothelium and hippocampus of APP/PS1 mice by immunohistochemistry shown in Figure 5A. No Aβ plaque deposition was observed in the cortex and hippocampus of the Control group mice. There were obvious Aβ plaques in the cortex and hippocampus of the model group. The deposits were denser, with clear boundaries, darker coloration, and large plaque areas. Compared with the model group, Aβ deposition in the cortex and hippocampus of the positive control group decreased. Three kinds of *Tieguanyin* extracts reduced the APP/PS1 mouse brain plaque deposits compared with the model group, and the amount and area of Aβ plaque deposits in the mouse brain were reduced to varying degrees compared with the model group (Figure 5B–E). There is no significant difference between *Tieguanyin* extracts group and Donepezil group, indicating that *Tieguanyin* extracts and Donepeizil have an ameliorating effect on amyloid plaque deposition, which suggests that *Tieguanyin* extracts are effective in restoring brain damage, cognition, and behavior in mice. The above results indicate that *Tieguanyin* extracts can reduce the deposition of Aβ plaques in the cortex and hippocampus of APP/PS1 mice, and reduce the pathological damage caused by Aβ deposition.

### 2.6. Tieguanyin Extracts Improves Brain Oxidative Stress in APP/PS1 Mice

**Figure 6:**
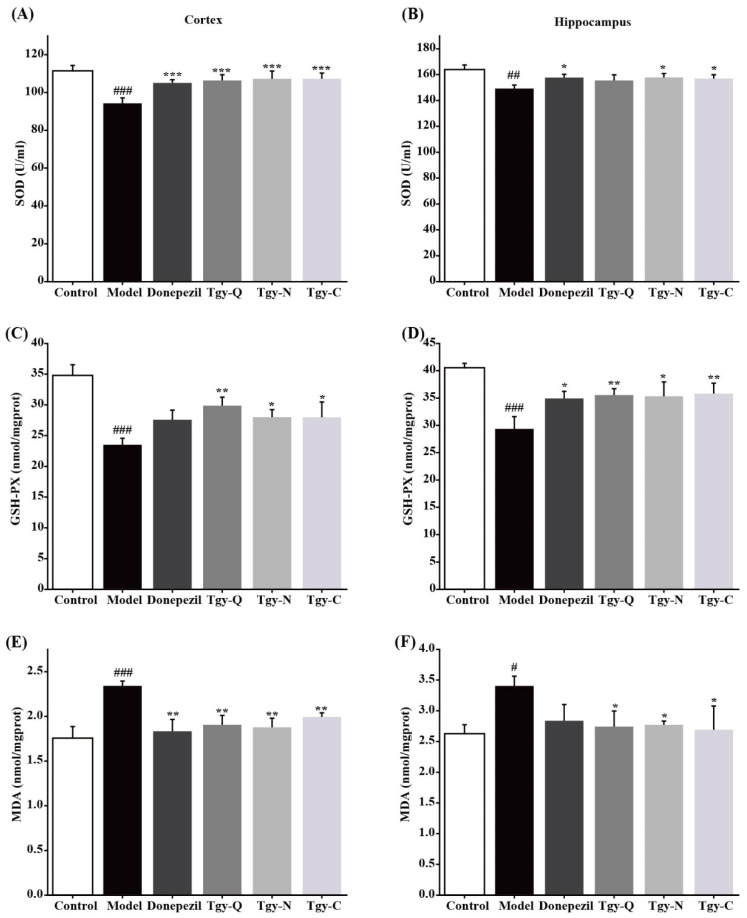
Anti-oxidative abilities of *Tieguanyin* tea extracts on cortex and hippocampus of mice in different groups. (**A**,**C**,**E**) Changes of SOD, GSH-PX, and MDA in cortex. (**B**,**D**,**F**) Changes of SOD, GSH-PX, and MDA in hippocampus) (Control group *n* = 7, *n* = 5 for each other group). # *p* < 0.05, ## *p* < 0.01, and ### *p* < 0.001 different from control group. * *p* < 0.05, ** *p* < 0.01, and *** *p* < 0.001 different from model group.

Aβ causes oxidative stress hierarchical reactions, these reactions are also positively related to the synthesis and deposition of Aβ We further tested the effect of *Tieguanyin* extracts on oxidative stress in the cerebral cortex and hippocampus of APP/PS1 mice, and found that compared with the blank group, the MDA level in the cortex of the model group had significantly increased, and the SOD activity and GSH-Px levels had decreased significantly. Compared with the model group, the MDA level in the mouse cortex of the Donepezil group was significantly reduced, and the SOD activity and GSH-Px level were significantly increased; while the MDA level in the cortex of mice in the Tgy-Q, Tgy-N, and Tgy-C groups was significantly reduced, SOD activity and GSH-Px levels were significantly increased (Figure 6A,C,E). Similar results were observed in the hippocampus (Figure 6B,D,F). The above results demonstrate that *Tieguanyin* extracts can significantly reduce the level of oxidative stress in the cerebral cortex and hippocampus of APP/PS1 mice.

### 2.7. Tieguanyin Extracts Reduces Neuroinflammation in the Brain of APP/PS1 Mice

**Figure 7:**
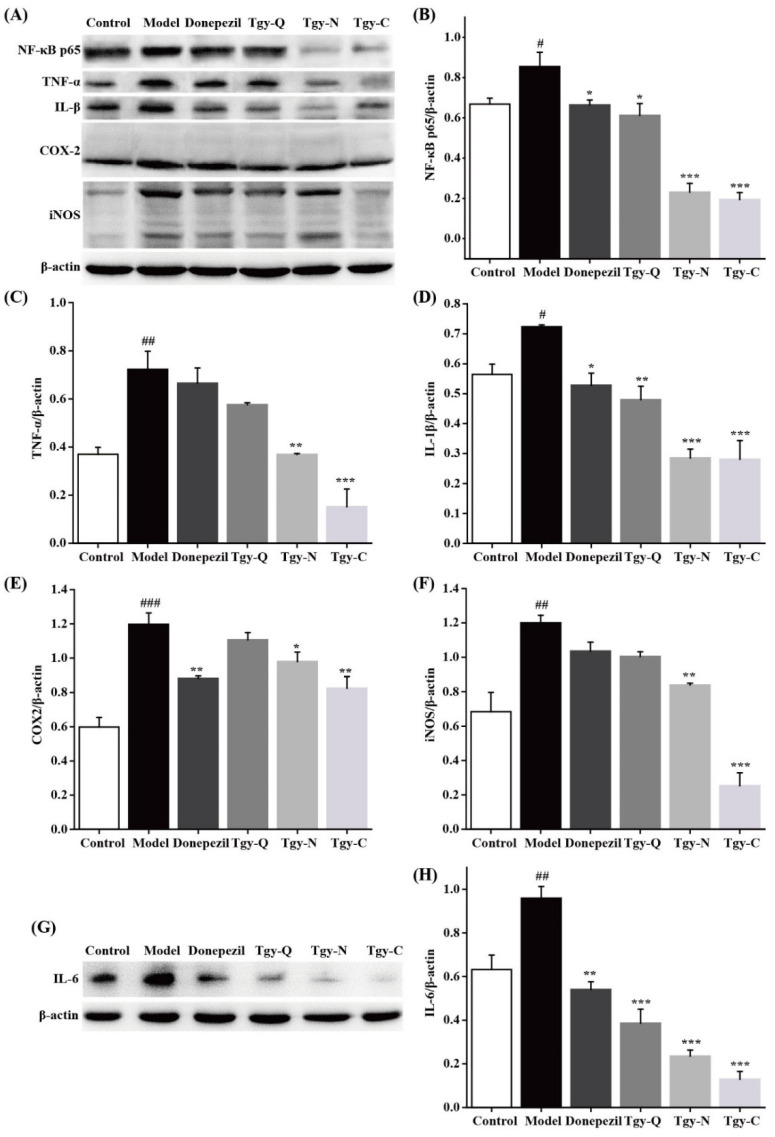
*Tiguanyin* extracts reduces the expression of inflammation related proteins in microglia. (**A**–**F**) Expression of NF-κB, TNF-α, IL-1β, COX-2, and iNOS in microglia in different groups. (**G**,**H**) Expression of IL-6 in microglia in different groups. Control group *n* = 7, *n* = 5 for each other group. Data are mean ± SEM. # *p* < 0.05, ## *p* < 0.01, and ### *p* < 0.001 different from control. * *p* < 0.05, ** *p* < 0.01, and *** *p* < 0.001 different from model group.

As an inflammatory stimulating factor, Aβ can stimulate the activation of cells and cause the release of inflammatory factors [13,14]. In addition, neuroinflammation is accompanied by the entire process of AD occurrence and development. Abnormally activated microglia and a large amount of inflammatory factors can jointly trigger neuroinflammation in experimental animals [14,15]. In our study, as shown in Figure 7, we found that compared with the blank group, the protein levels of NF-κB p65, TNF-α, IL-1β, and IL-6 in the brain tissue of the model group increased significantly. Compared with the model group, the levels of NF-κB, IL-1β, and IL-6 in the Donepezil group decreased significantly, and there was no significant performance difference in TNF-α. The levels of NF-κB, IL-1β, and IL-6 in the Tgy-Q group decreased significantly, but there was no significant difference in TNF-α, NF-κB, TNF-α, IL-1β, and IL-6 in the Tgy-N, and the Tgy-C groups decreased significantly. Compared with the control group, the COX-2 and iNOS protein levels in the brain tissue of the model group increased significantly, and compared with the model group, there was no significant difference in COX-2 and iNOS in the Tgy-Q group, and IL-6 was significantly reduced, while the COX-2, iNOS, and IL-6 in the Tgy-N and Tgy-C groups were significantly reduced. The above results demonstrated that *Tieguanyin* extracts could significantly down-regulate the expression of inflammatory related proteins in brain, and finally alleviate the AD syndromes in APP/PS1 mice.

## 3. Discussion

Tea has been evidenced to have antioxidant, neuroprotective, and anti-aging effects. Aging, oxidative stress, nerve damage, and even neuronal apoptosis caused by various reasons are all key factors in the development of neurodegenerative diseases. For the first time this paper explore the effects and mechanisms of Tgy-Q, Tgy-N, and Tgy-C extracts prepared with different processing methods from the same raw material on AD. The three types of *Tieguanyin* extracts all contain high concentrations of bioactive ingredients such as tea polyphenols, amino acids, soluble proteins, soluble sugars, tea polysaccharides, flavonoids, and caffeine. In this study, we found that *Tieguanyin* extracts can significantly improve the cognitive function, learning and memory ability of APP/PS1 mice by targeting the inflammation in brain.

The hippocampus is an important area for learning and memory, and the damage of neurons in the hippocampus is closely related to the learning and memory function [16]. Neurons in the hippocampus of APP/PS1 mice is obviously damaged [17], thus this mouse module is very suitable for studying the anti-Alzheimer effect of natural products. Under normal circumstances, Aβ is continuously degraded and quickly cleared in the mice brain, and the process of its production, decomposition, and transport maintains a dynamic balance without forming deposits [17]. However, in pathological conditions, abnormal deposition of Aβ can cause changes in oxygen free radical levels, destruction of cell membrane structure, lipid peroxidation, induction of cell apoptosis, and chronic inflammation [18]. Aβ deposition can promote the release of inflammatory factors, which can damage learning and memory, and cause a series of neurological damage [19]. In this study we found that the three *Tieguanyin* extracts significantly improved brain plaque deposition in APP/PS1 mice and significantly reduced the contents of Aβ1-40 and Aβ1-42 in the mouse cortex and hippocampus. Our research is consistent with previous reports to a certain extent that EGCG in green tea can reduce Aβ production in APP mice and reduce amyloid plaques [20]. For the clinical drug aducanumab, which was approved to be used for the treatment of AD clinically in 2021, the effective dose in animal model could be as low as 3 mg/kg [21], although in our study, we used a much higher dose of *Tieguanyin* extracts, our results could provide evidence that the beneficial effects of drinking tea in the prevention of AD.

Oxidative stress means that under normal conditions, the body’s oxidation and anti-oxidation are in a balanced state. When there are too many oxidation products, oxidative stress will occur. MDA, GSH-Px, and SOD are important indicators for the evaluation of oxidative stress. We found that three *Tieguanyin* extracts significantly reduced the MDA content in the APP/PS1 mouse brain, increased SOD activity and GSH-Px activity, which suggests that the three *Tieguanyin* extracts can reduce oxidative stress levels in the brains of APP/PS1 mice by improving endogenous antioxidant capacity and reducing peroxide levels. A previous study compared the neuroprotective effects of green, red, and black tea, and found that green tea supplementation demonstrated the best effect in an Alzheimer-like rat model [9]. Recent research demonstrated that green tea extracts could significantly suppress amyloid β levels and alleviate cognitive impairment at a daily consumption of 700 mg/kg in a mouse model [22]. Our finding is consistent with these results. The reason why green tea has the most efficient effect may be due to its higher catechin content [23,24], and the three *Tieguanyin* tea extracts used in this study contains high amounts of catechin. The differences of extracts as well as the specific role and contribution of catechin need and are worth of further study in our future work.

A large amount of ROS generated under oxidative stress will cause the activation of microglia, which exerts nerves by releasing large amounts of nitric oxide (NO), prostaglandin E2 (PGE2), iNOS, COX-2, TNF-α and other toxic factors, which finally leading to inflammation. In this process, the NF-κB signaling pathway plays a vital role. The NF-κB signaling pathway is closely related to the deposition of Aβ in the AD brain, as well as neuroinflammation and oxidative stress [25]. NF-κB is involved in a variety of biological responses such as neurotransmission, immune inflammation, cell growth, and gene regulation [26]. Inflammation and oxidative stress are closely related. The appropriate activation of NF-κB plays an important role in the body damage caused by various factors, However, its excessive activation can lead to the expression and release of inflammatory factors IL-1β, IL-6, etc., and aggravate pathological reactions. Catechin, the main active ingredient in tea, has anti-oxidation, anti-inflammatory, and cholinesterase activity inhibition, and can prevent AD. Studies have found that EGCG in tea can inhibit the activation of astrocytes and the TLR4/NF-κB pathway can improve cognitive ability in APP/PS1 mice [27]. The three *Tieguanyin* extracts used in this article contained a large amount of catechins including EGCG, and it was found that these three *Tieguanyin* extracts significantly improved the learning and memory ability of APP/PS1 mice and decreased the deposition of Aβ plaques. The mechanism of action was further explored by detecting NF-κB pathway related proteins, and the results confirmed that the three *Tieguanyin* extracts can down-regulate inflammatory factors such as NF-κB p65, TNF-α, IL-1β, IL-6, COX-2, iNOS, etc., thereby alleviating the symptoms of AD of APP/PS1 mice. The specific role of NF-κB p65 signaling pathway in the alleviating of AD symptoms by *Tieguanyin* extracts is worthy of in-depth study.

## 4. Materials and Methods

### 4.1. Materials and Chemicals

Tgy-Q, Tgy-N, and Tgy-C prepared by identical raw materials were manufactured by Fujian Anxi Qishan Weiyin Famous Tea Co., Ltd., and kindly provided by Mr. Yuede Wei, who oversaw the manufacturing processes of the experimental tea products for this study. Brief processing of these three products was as following: the raw leaves (one bud and three or four leaves) for making these three types of *Tieguanyin* tea were hand-picked from cultivated *Tieguanyin* cultivar at the end of September. The authenticity of *Tieguanyin* cultivar was verified by professor Jiangfan Yang. The leaves then underwent withering, shaking, fixation, rolling, and drying. The major difference for these three types of *Tieguanyin* products are driven by the shaking step and the following storage. Detailed manufacturing processes of these three products was shown in Figure 1. Standard catechins (–)-gallocatechin (GC), (–)-epigallocatechin (EGC), (+)-catechin (C), (–)-epigallocatechin-3-gallate (EGCG), (–)catechin gallate (CG), (–)gallocatechin gallate (GCG), (–)epicatechin (EC), (–)epicatechin gallate (ECG), gallic acid (GA), and Caffeine were purchased from Sigma-Aldrich (St. Louis, MO, USA).

### 4.2. Preparation of Tieguanyin Extracts

Three types of *Tieguanyin* samples were powdered and extracted with boiling water for 30 min, respectively, then the filtrate was collected after vacuum filtration. The residue was re-extracted with boiling water for 20 min. Filtrates were combined and concentrated with rotary evaporator to about one-tenth of original volume, the concentrated solution was stored at −20 °C refrigerator for 2 h and then freeze-dried to make *Tieguanyin* extracts. The extracted were packaged in the refrigerator at −20 °C before use.

### 4.3. Determination of Chemical Components of Tieguanyin Extracts

Tea polyphenols and flavonoids were measured according to the method described by Xu et al. The content of tea polyphenols was conducted by the Folin method: 1.0 mL test solution was transferred in the tube, then 5.0 mL 10% Folin reagent was added, then mixed and reacted for 5 min. A total of 4.0 mL 7.5 NaCO_3_ was added and placed in the reaction tube at room temperature for 60 min, and the absorbance was measured at 756 nm. The content of polyphenol was calculated according to the standard curve. The content of flavonoids was determined with minor modifications, briefly, aluminum trichloride colorimetric method was used after adding 10 mL (1% aluminum trichloride) aqueous solution to 0.5 mL of sample solution, shaken well, and rested for 10 min for through reaction. A solution of 1% aluminum trichloride was used as a blank, absorbance was measured at 420 nm, and the calculation was performed by the comparing the absorbance of flavonoid glycosides control sample [10]. Soluble sugar was quantified with the sulfuric acid-anthrone colorimetric method [28]. Caffeine, GA and tea catechins were analyzed with high-performance liquid chromatography (HPLC) following the method described by Tai et al. with modifications [29], briefly, a Shimadzu LC-2010HT high-pressure liquid chromatography (Shimadzu, Japan) was used which equipped with Agilent TC-C18 reversed-phase column (150 mm × 4.6 mm, 5 μm) with the UV spectra was set at 280 nm and column temperature at 35 C. Gradient elution with distilled water/acetonitrile/acetic acid (96.5:3:0.5, *v/v/v*) (A) and distilled water/acetonitrile/acetic acid (69.5:30:0.5, *v/v/v*) (B) at a flow rate of 1 mL/min from 30% solvent B to 80% solvent B in 35 min and then back to 30% solvent B in 5 min. Total free amino acids were measured following the method by Jabeen et al. [30]. The content of tea polysaccharides was determined by the anthrone-sulfuric acid method with minor modifications [31]. The content of soluble protein was determined by Coomassie Brilliant Blue method [32].

### 4.4. Animals

Adult mice (males, 6 months) were used for behavior and biochemical experiments. 40 APP/PS1 (APPswe, PSEN1dE9) mice with a C57BL/6J background and 10 C57BL/6J mice were purchased from Shanghai Model Organisms Center, Inc. (Shanghai, China) (Mice originated from Jackson Lab, Bar Harbor, ME, USA) and maintained in the Experimental Animal Center of Zhejiang Chinese Medical University (ZCMU) which located in Hangzhou, China. All the animal protocols have been reviewed and approved by Laboratory animal management and ethics committee of ZCMU with approval No. IACUC-20190729-09. Mice were housed under a 12-h light/12-h dark cycle with access to food and water. All procedures were approved and strictly follow the guidelines of Zhejiang Provincial Animal Ethics Association. In our study, the dose we chose was 1000 mg/kg. The dose is equivalent to (1000/10 × 60 = 6 g) *Tieguanyin* extract, which is about 18 g *Tieguanyin* tea for a 60 kg human. The extracts were dissolved with purified water at a concentration of 10 mg/mL, each mouse was administrated 0.25 mL by oral gavage. Mice were randomly assigned to 6 experimental groups; (1) control (10 mice, C57BL/6J), (2) model (8 mice, APP/PS1), (3) Donepezil (8 mice, APP/PS1, 1 mg/kg/d), (4) Tgy-Q (8 mice, APP/PS1, 1000 mg/kg/d), (5) Tgy-N (8 mice, APP/PS1, 1000 mg/kg/d), (6) Tgy-C (8 mice, APP/PS1, 1000 mg/kg/d). Different doses of vehicle or *Tieguanyin* extracts were administrated by daily oral gavage at a volume of 0.25 mL for 70 days. All mice behavioral procedures were double blinded.

### 4.5. Morris Water Maze Test

The Morris water maze (Smart, Panlab SL, Barcelona, Spain) device with a diameter of 100 cm and a circular pool of 70 cm high was used. The depth of the pool is 30–40 cm with the water dyed with edible iron white powder to make sure the hidden platform was not visible to the mice. Water temperature was controlled at 22~25 °C. A round platform with a diameter of about 9 cm was set up and the white platform was hidden 1 cm below the water surface to ensure that the mouse cannot see the platform when swimming. An overhead video camera connected to the tracking software was used for the study. Each trial was conducted for 60 s until the mouse found and climbed onto the white platform. Data analysis was conducted following the described method [33].

### 4.6. Novel Object Recognition Test

Novel object recognition assay was conducted following the described method [34]. Briefly, a square open field detection box (length 50 cm, width 50 cm, height 40 cm) was used. The infrared camera tracking system was connected to a computer and supporting software. Cubes (5 cm × 5 cm × 5 cm) and cylinders (diameter 5 cm × height 5 cm) were used as recognized objects. The mouse was put into the experimental field and explored freely for 5 min to adapt to the environment 24 h before the experiment. Two identical objects (A1, A2) were placed on the left and right ends of a side wall, which were parallel to the wall and equidistant from the surroundings. After putting the mouse in the detection box, they were recorded for 5 min. New object recognition and detection was performed after 3 h by replacing A2 with a new object, then the mouse was put back in again and recording for 5 min. The time spent on exploring the new object within 3 cm from the it and the percentage of time spent exploring new objects in total time were calculated.

### 4.7. Immunohistochemistry

After the end of the behavioral experiment test, all the experimental mice were fasted for 12 h. On the tissue collection day, the mice were injected with sodium pentobarbital (50 mg/kg) for anesthesia. Then, they were perfused with cold PBS and 4% paraformaldehyde. Mice brains were immersion-fixed in 4% paraformaldehyde. Brain tissues were trimmed, processed, and stained for Nissl, Aβ1-40, and Aβ1-40.

### 4.8. Hematoxylin and Eosin (H&E) Staining

H&E staining was performed based on previous protocol [35]. Briefly, the fixed brain tissue was cut into 3.5 μm brain tissue, dewaxed, stained with hematoxylin, dehydrated, stained with eosin, washed, and transparentized. After mounting, the mouse cerebral cortex and hippocampus (CA1, CA3, CA1, CA3, DG) morphology and structure, and images were captured for further analysis.

### 4.9. Quantification of Aβ1-40 and Aβ1-42 in Brain Tissues by Elisa

After the end of the behavioral experiment test, all the experimental mice were fasted for 12 h. On the tissue collection day, the mice were injected with sodium pentobarbital (50 mg/kg, i.p.) in the abdominal cavity for anesthesia. Subconjunctival hemorrhages were performed for blood collection and following this the heads were decapitated. The cerebral cortex and bilateral hippocampus tissues were quickly removed and placed in liquid nitrogen. After the completion of the collection, they were quickly transferred in refrigerator at −80 °C, before the determination of antioxidant activities, enzyme-linked immunosorbent assay, and western blotting. The contents of Aβ1-40 and Aβ1-42 in brain tissue were determined according to the manufacturer’s protocol. Briefly, brain tissue was homogenized on ice, then the homogenate was centrifuged at 5000× *g* for 10 min at 4 °C, and then 10% of mouse brain homogenate supernatant was taken for detection of Aβ1-40 and Aβ1-42 contents. First, 100 μL of each sample was added to each well, then the ELISA plate was covered and placed in a 37 °C incubator for 90 min, before the liquid in the well was drained off, and 100 μL of biotinylated antibody working solution was added to each well. The membrane was incubated for 1 h at 37 °C, the liquid in the wells was shaken off, 350 μL of washing solution was added to each well, and then the membrane was soaked for 2 min, and repeated three times. A total of 100 μL of enzyme conjugate working solution was added to each well, incubated at 37 °C for 30 min and then washed five times, before 90 μL of substrate solution (TMB) was added, incubated at 37 °C incubator for 15 min, and finally 50 μL of stop solution was added, before the optical density was immediately measured at 450 nm wavelength.

### 4.10. Western Blotting

Western blotting was conducted following previous method [36]. Briefly, the brain tissue was lysed and centrifuged to take the supernatant, the protein concentration was determined by the BCA method. Samples were mixed with 5 × sodium dodecyl sulfate loading buffer, and after boiling for 10 min, the same amount of protein was loaded on to a 5% gradient polyacrylamide-SDS gel. Then, the proteins were transferred to a PVDF membrane for 1 h at 100 V. Then, after being blocked with 5% milk-PBST for 1 h, the samples were incubated with primary antibodies overnight. Membranes were washed with PBST five times, and then incubated with secondary antibody for 1 h in PBST at room temperature. ECL reagent was used for imaging. β-actin was used as internal control.

### 4.11. Statistical Analysis

All data were expressed as mean ± standard error of mean (SEM) from three replicates. Microsoft Office Excel 2019 and SPSS Statistics version 22.0 (IBM, Incorporation, Chicago, IL, USA) were used for statistical analysis. One-way analysis of variance (ANOVA) and Tukey’s test were used for significant differences analysis with statistical significance was set to *p* < 0.05. Origin software 8.0 was used for illustrating. Control vs. model uses # to indicate significant difference, model vs. Tgy-Q, Tgy-N and Tgy-C uses * to indicate significant difference. ^#/^* *p* < 0.05, ^##/^** *p* < 0.01, and ^###/^*** *p* < 0.001 indicate statistical differences.

## 5. Conclusions

In summary, we proved for the first time that the extracts of *Tieguanyin* made from the same raw material but with different processing technologies can significantly improve the cognitive ability of APP/PS1 mice. *Tieguanyin* extracts can effectively reduce Aβ deposition in the brain of mice and regulate the level of oxidative stress in the brain, regulating the NF-κB signaling pathway and alleviating the neuroinflammatory response caused by Aβ deposition. This thereby reduces the activation of NF-κB p65 protein, down-regulates the expression of COX-2, iNOS proteins and the expression of TNF-α, IL-1β, and IL-6 pro-inflammatory factors, thus alleviating the syndromes of AD in APP/PS1 mice. The empirical findings in this study provide a new understanding of the beneficial effect of *Tieguanyin* tea in the context of alleviating neurodegenerative diseases; however, more work is needed for understanding the health benefits of *Tieguanyin* tea.

## Figures and Tables

**Table 1:** Major chemical constituents in the Tgy-Q, Tgy-N and Tgy-C extracts.

Chemical Constituents	Tgy-Q	Tgy-N	Tgy-C
Tea polyphenol (mg/g)	437.25 ± 2.13 a	396.48 ± 3.81 b	368.26 ± 2.92 c
Amino acids (mg/g)	64.08 ± 0.60 a	62.55 ± 0.74 b	47.92 ± 0.28 c
Soluble proteins (mg/g)	88.61 ± 3.01 b	92.11 ± 2.79 b	104.73 ± 5.25 a
Soluble sugars (mg/g)	362.01 ± 8.19 a	336.52 ± 4.35 b	278.18± 4.17 c
Tea polysaccharides (mg/g)	48.64 ± 2.11 b	37.29 ± 1.00 c	57.35 ± 1.52 a
Flavonoids (mg/g)	24.18 ± 0.54 b	26.56 ± 1.78 b	32.71 ± 0.97 a
Caffeine (mg/g)	71.36 ± 0.89 b	77.12 ± 0.73 c	77.49 ± 0.92 a
Gallic acid (mg/g)	2.14 ± 0.21 c	3.76 ± 0.17 b	6.67 ± 0.44 a
Gallocatechin (mg/g)	38.37 ± 1.08 a	30.35 ± 1.62 c	30.39 ± 1.76 b
Epigallocatechin (mg/g)	49.06 ± 1.86 a	45.80 ± 1.14 b	38.47 ± 0.42 c
Catechin (mg/g)	7.74 ± 1.61 a	8.12 ± 0.76 a	8.70 ± 1.72 a
Epicatechin (mg/g)	24.59 ± 1.42 a	16.32 ± 0.77 b	5.41 ± 0.44 c
Epigallocatechin gallate (mg/g)	105.21 ± 1.64 a	86.99 ± 1.77 b	77.42 ± 0.91 c
Gallocatechin gallate (mg/g)	31.24 ± 1.07 b	32.54 ± 1.29 b	36.12 ± 1.31 a
Epicatechin gallate (mg/g)	32.05 ± 0.84 a	30.74 ± 1.34 a	24.83 ± 1.24 b
Catechin gallate (mg/g)	6.28 ± 0.64 a	6.48 ± 1.47 a	6.415 ± 1.52 a
Total catechins (mg/g)	294.56 ± 10.91 a	257.35 ± 10.20 b	227.79 ± 9.36 c

Data represent mean ± SD of three replicates. Different lowercase letters in the same row indicate significant differences between mean values (*p* < 0.05).

## Data Availability

The data presented in this study are available on request from the corresponding author Youying Tu.
